# Washington County Medical Society

**Published:** 1886-02

**Authors:** 


AND ANOTHER.—We are much gratified to learn as we do through our much esteemed friend (and subscriber, of course) Dr. S. Farrar Styles, of Independence, that the Washington County Medical Society, which has been under the somniferous influence, seemingly, of some mancanilla tree, and, consequently, sleeping, has caught the slogan note of reform, and has again taken up its line of march in the grand procession now in motion throughout Texas.

There is a thorough awakening all along the lines of the medical profession to the *necessity* of concert of thought and action in matters of vital importance to the profession, as well as to the people. There *must be a reform, a purifaction*, and advance in Texas medicine! The people of Texas must know *who are and who are not physicians*, entitled, by their *training, qualification* and education, to the *right* to practice medicine, and to their confidence—usurpers of the high and responsible prerogative—those fellows who have, to paraphrase Pollock—"stolen the livery of medicine to serve the devil in"—must "get further"—or subside. It is time that medical men were asserting *their rights*, and, amongst those rights, to be protected, to some extent, from bastard competition is one of much importance. The lamented Bowling is often quoted as saying, "To medical men belong medical matters." Well, if medical practice is not a medical matter, what is? The right to practice medicine *should be* restricted to the hands of those who have been trained and educated to the calling; it matters not how or where, so they *are* trained and educated. In the hands of *any other*, it is a privilege more deadly than the Gatlin gun, or of nitro-glycerine. Another very important right is that of experts, or medical witnesses, before courts.

But—hello here! we have flown the track, and, under the head of "Society Notes," we had switched on to the editorial sideling, and were going at a fearful speed. We will run into the round-house, blow off steam and subside—and then back out on to the main track, open the throttle, and go ahead again on the Washington County Medical Society's revivification! Dr. Styles says:

"We got up a medical organization in March, and I was made President. We never succeeded in getting another meeting till last Tuesday, when we had quite an interesting one. Dr. Lockhart was the essayist, and he read a splendid paper on diphtheria. The discussion which followed was extensive, interesting and edifying. We think now there will be no further trouble about our meeting regularly in the future. The subject for our next is dengue, and I would not be surprised if you get a valuable paper on that subject after the meeting. We meet first Tuesday in each month. When we get under headway, you must come down and aid us with your presence and advice," etc.

